# Incorporation of Phase Change Materials into the Surface of Aluminum Structures for Thermal Management

**DOI:** 10.3390/ma15196691

**Published:** 2022-09-27

**Authors:** Christopher Anderson, Forest Shaner, Walter Smith, Claudia Luhrs

**Affiliations:** 1 Department of Mechanical and Aerospace Engineering, Naval Postgraduate School, Monterey, CA 93943, USA; 2 Veterans for Energy Careers Intern, Naval Postgraduate School, Monterey, CA 93943, USA

**Keywords:** phase change material, PCM, latent energy storage, eicosane, anodization techniques, passive thermal management, transient thermal loads

## Abstract

This article explores the concept of generating a porous anodic layer on the surface of a metallic component to host a phase change material (PCM) aiming to reduce the peak temperatures that the host structure will experience. The conditions to fabricate a porous anodic layer on top of an aluminum substrate were determined through varying anodization conditions: solution concentration, voltage employed, and anodization times. Pore sizes were characterized using scanning electron microscopy. The alkane n-eicosane was selected as PCM, introduced within the porous anodic annealed layer using vacuum impregnation and the thin film composite structure sealed. Epoxy resin and a metallic paste were tested as sealants. Thermal tests were performed to compare the behavior of aluminum alloy substrates anodized and sealed with and without PCM. The results showed pores with diameters in the 5–85 nm range, with average values that increased as the time of anodization was extended. The aluminum alloy impregnated with n-eicosane presents lowered surface peak temperatures during heating cycles than the samples that were only anodized or than the base alloy, demonstrating the potential of PCM incorporated in the superficial microstructure of anodic structures to manage, to a certain extent, peak transient thermal loads.

## 1. Introduction

Multiple systems and components operate at high power, high speed, or under circumstances that expose them to elevated temperatures, all of which could compromise their functionality. Thermal runaway in batteries, central processing unit (CPU) overheating, and ablation of materials are common examples of the negative effects that high temperatures can cause. To protect equipment from thermal damage, various types of thermal management systems have been developed, which can often be categorized into active and passive thermal management. An example of active thermal management is a refrigeration system that pumps refrigerant to absorb heat from one side of the system and expels heat at a different site; that is, the active thermal management system uses equipment to transport fluid that carries heat from the component to the environment. Such is the case with some refrigeration systems in DDG-51 class naval ships, which employ R-114 refrigerant [1]. Overall, active thermal management strategies are very effective means of reducing the temperature in a system; however, those require different designs and additional power than passive management approaches, which result in higher operating costs [2]. To cut down on energy costs, industries have been investing in passive cooling systems, which employ materials and/or geometries applied to equipment to effectively exchange heat with the environment without the need for a power source. Heat sinks, heat spreaders, and heat pipes are all examples of passive thermal management. Phase change materials (PCM) are another example of a passive thermal management approach, which could be found in applications related to electronics, fabrics, cold storage, and medical devices [3,4,5,6,7,8]. PCMs absorb and release heat at temperatures at or near their phase change temperature. These phase changes are endothermic while heating; when the material is transitioning from solid to liquid, and exothermic upon cooling; during the transformation from liquid back to solid. Furthermore, PCMs have been successfully employed by diverse industries, including solar [9], buildings/construction [10], textiles [8], and electronics [11]. Organic compounds stand out as phase change materials when compared to their inorganic counterparts due to their high latent heat values, low-cost, stability over a large number of cycles, and availability [12,13,14].

Despite PCM usage within many passive thermal management system applications, the use of PCM introduced in metallic structures at the microstructural surface level, to the best of our knowledge, has not been sufficiently explored. Thus, the goal of this study was to engineer a porous surface layer that incorporated an organic PCM in a metallic microstructure (aluminum alloy) using a simple post-production process that could be applied to existing, geometrically complex metal parts. To accomplish this, a fabrication route able to generate porous structures without damaging the host structure or reducing its mechanical properties had to be identified. The desired microchannels or pores would need to be able to accept the phase change material and be strong enough to sustain the various forces placed upon them. Moreover, the PCM will need to be sealed to prevent it from leaking during the heating stages and be able to sustain multiple thermal cycles. The sections below describe the use of anodization as the means to produce the porous surfaces, vacuum impregnation as the process employed to introduce the PCM in the channels, and the use of two different sealing materials. The manuscript proceeds then to quantify the effect of introducing PCM into the surface of an aluminum fin similar to those employed in commercial heat sinks, and the newly generated composite surface layer’s ability to absorb heat and maintain lower peak temperatures during transient thermal loads than those devoid of PCM.

## 2. Materials and Methods

### 2.1. Surface Layer Fabrication

**Figure 1:**
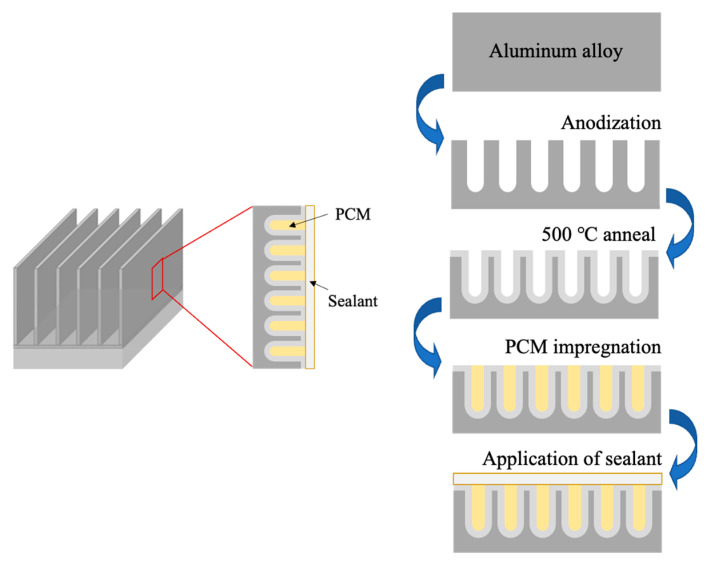
Schematic representation of the steps followed to generate a porous structure on the surface of a metallic component, anneal it, introduce the PCM, and seal it, to produce a metal-PCM composite at the microstructural level in the surface layer of the base alloy.

Instead of starting with the full body of a heat sink, this study employed an aluminum alloy fin that was removed from a commercial heat sink to serve as proof of concept that could later be scaled up. Figure 1 illustrates the steps employed to generate the composite structure on the surface of the metallic host. The aluminum fin was anodized to create a porous layer, annealed at moderate temperatures to strengthen the anodic structure, vacuum impregnated with n-eicosane PCM, and sealed with either epoxy resin or silver paste. Eicosane was selected due to having high latent heat energy of 237 kJ/kg. Other properties include transformation temperatures between 36 and 39 °C, density at 0.7889 g/cm^3^, and being widely available from commercial sources.

**Figure 2:**
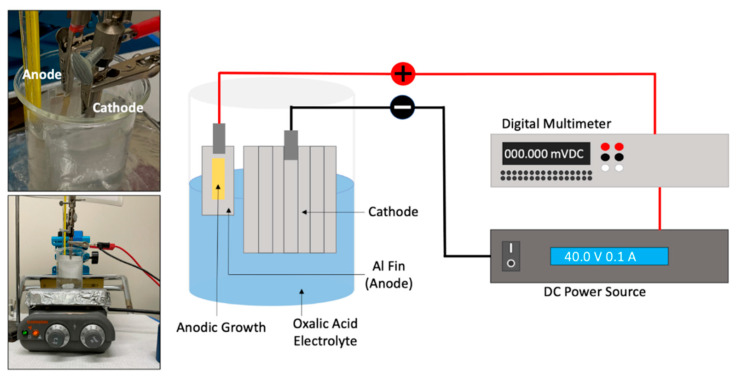
Experimental setup used to produce an anodic porous structure on the surface of the aluminum alloy sample. From left to right: oxalic acid bath containing anode and cathode along the hot plate used to maintain bath at a given temperature, and representation of the connections from the anode and cathode to DC power source and multimeter.

All samples were anodized with the setup shown in Figure 2. The aluminum alloy sample to be anodized was employed as the anode while the cropped aluminum heat sink base of the same alloy was used as a cathode. The cathode had a surface area that was significantly larger than that of the anode (1:32 ratio). Electrical insulation tape was used to cover the back and the edges of the aluminum fin anode, leaving approximately 120 mm^2^ of aluminum exposed to the electrolyte. A DC power source (Model XLN15101 B&K Precision Corp, Yorba Linda, CA, USA) and a digital multimeter (Model 2100 Keithley Instruments, Cleveland, OH, USA) were placed in series and connected to metal clamps that held the anode and cathode; the positive end was connected to the anode and the negative end to the cathode. The power source was programmed to supply a fixed amount of voltage over a set amount of time.

Concerning the anodization conditions, the method was adopted from a study conducted by Sanz et al. [15]. For the purposes of this study, well-structured open pores of micrometer scale length were sought after to host the PCM. The aluminum alloy was anodized at 1.2 and 1.6 M oxalic acid (98%, Sigma-Aldrich, Burlington, MA, USA) concentrations, using voltages of 20, 30, 40, and 100 V, with a fixed value of 40 °C for the bath temperature. The experiments were performed during time intervals that spanned between 10 and 40 min.

After the creation of the anodic layer, the tape employed to limit the area exposed to the bath was removed from the fin samples, which were then rinsed with DI water, ethanol, and then dried. The specimens were placed in an oven at 500 °C for 2 h (process herein referred to as anneal) and allowed to gradually cool.

**Figure 3:**
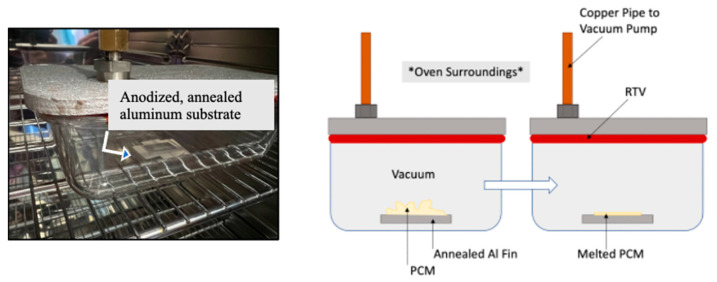
Vacuum impregnation of PCM into porous anodic layer inside an oven. Left: Picture of the sample, location indicated by arrow, inside a hermetically sealed container connected to a vacuum pump while heated inside an oven. Right: Schematic representation of the PCM melting under vacuum to produce a thin layer with standard air designated by the note *Oven Surroundings*.

During the vacuum impregnation process, the non-anodic sections of the aluminum fin were again covered in tape, and 0.05 g of solid n-Eicosane (Millipore Sigma, Saint Louis, MO, USA) was placed on top of the anodic layer generated at 40 V, 1.6 M at 40 °C for 25 min. The fin was then placed in a hermetically sealed dish that was connected to a vacuum pump (roughing pump that achieves 1 × 10^−3^ torr) and placed into a binder convection oven (Binder GmbH, Tuttlingen, Germany). Once the vacuum pump was activated, the oven was set to 45 °C for the PCM to melt. The intent of placing the liquid PCM below atmospheric pressure was to force the PCM to fill the vertically oriented pores that the anodization step produced. Samples were allowed to cool while under vacuum. See Figure 3.

To prevent phase change material leakage during the heating and cooling cycles, each sample was sealed by the application of a surface coat. Diverse sealing materials were compared based on their availability, ease of application, thermal stability, and the ability to contain the PCM after several heating cycles. To prepare each sample, the outer rim of the aluminum fin was washed with ethanol to ensure that all tape residue from the vacuum impregnation process had been removed. The non-anodic surface was then sanded with 300-grit sandpaper to ensure that the chosen sealant had a strong mechanical bond to the area surrounding the phase change material.

Epoxy resin was used as one of the methods of encapsulation. Epofix resin (Struers, Ballerup, Denmark), with a 25 to 3 resin-to-hardener ratio by mass, was chosen for the first experiment. The resin–hardener mixture was stirred for five minutes and allowed to settle for five additional minutes, then a thin coat of epoxy was applied with a fine-haired brush. Once the fin had the uncured epoxy applied, it was placed in the same vacuum chamber used for impregnation to remove air bubbles introduced from mixing the epoxy components. Once the vacuum was applied using the roughing pump, the epoxy system was allowed to dry at room temperature for 24 h.

The second sealant used, here referred to as silver paint, was composed of silver particles dispersed in Iso-Butyl Methyl Ketone (Ted Pella, Inc., Redding, CA, US). To distribute the silver particles prior to application, the silver paint bottle was placed in an ultrasonic cleaner for ten minutes. After covering the fin with silver paint, the aluminum fin was placed in the vacuum chamber for thirty minutes. While the aluminum fin was drying in the vacuum chamber, the silver paint was placed in the sonication bath to ensure that any particles displaced after the first application were again evenly distributed. After thirty minutes, a second coat of silver paint was applied to the entire surface and placed in the vacuum chamber for thirty minutes, after which the vacuum pump was turned off and the fin was left to dry for 24 h.

### 2.2. Materials Characterization

The porous structures produced were observed employing scanning electron microscopy (SEM). A Zeiss Neon 40 (Carl Zeiss Inc., Thornwood, NY, USA) field emission SEM and a FEI Inspect 50 SEM (Field Electron and Ion Company, Hillsboro, OR, USA), both operating between 1 and 20 KV were used to characterize the pore sizes and anodic layer height. The software program Image J (National Institutes of Health, Bethesda, MD, USA) was used for the statistical analysis of pore sizes.

The thermal behavior of the PCM selected was characterized by employing a Netzsch STA 449 F3 Jupiter (±2% J/g, ±0.001 K). The specimens were exposed to an Ar/O2, 80%/20% atmosphere from 30 to 50 °C at a heating rate of 1 °C/min.

Once the PCM was sealed, thermal testing was conducted using a FLIR USETS320 thermal camera (FLIR Systems Inc., San Carlos, CA, USA). The thermal test was constructed by placing a Pyrex dish filled with sand on top of a hot plate and positioning the samples on top of the sand. The FLIR camera was set 65 mm above the sample surface. The sand bed was used to slow the heating rate, allowing the measurement of the local temperatures within each sample. A bare aluminum fin, along anodized–annealed–sealed fins with, and without PCM, were tested simultaneously. The samples were sealed under identical conditions to match their emissivity. Heating and cooling cycles were run, and the temperature was plotted with respect to time for each sample.

## 3. Results and Discussion

**Figure 4:**
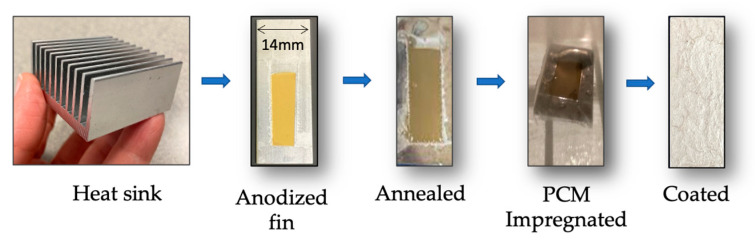
From left to right: Commercial aluminum heat sink employed; single fins were used for the initial proof of concept. The aluminum surface presented different surface finish and color as the samples undergo anodization, temperature treatment at 500 degrees C, were impregnated with PCM, and finally coated.

The outcome of the steps taken to generate the samples outlined in Figure 1 can be seen in Figure 4 below. Visual observation of the fin samples showed a yellow deposit after anodization, which turned brown after thermal treatments at 500 °C. The melting and dispersion of the PCM during vacuum impregnation left a thin film that was later coated. In Figure 4, the silver sealant is observed. Other specimens showed a transparent coat (epoxy resin) that allowed the observation of the underlying structure.

**Figure 5:**
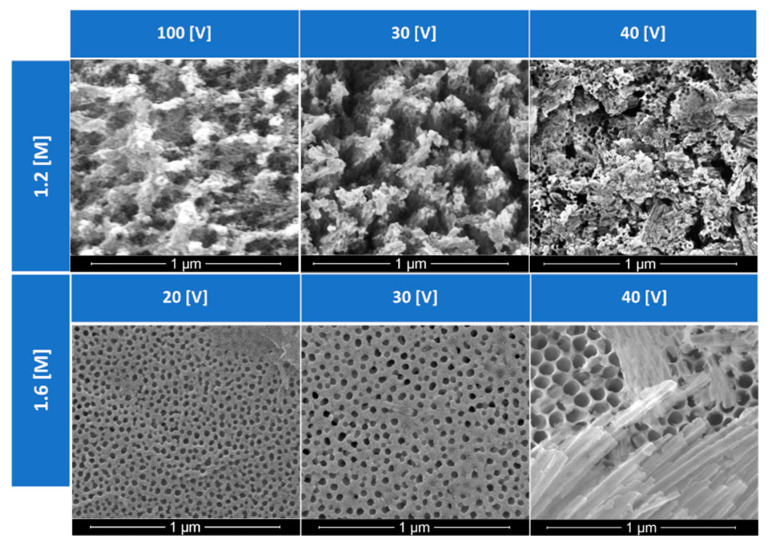
Effects of concentration and anodization voltage in the pore structure generated in the anodized aluminum.

**Figure 6:**
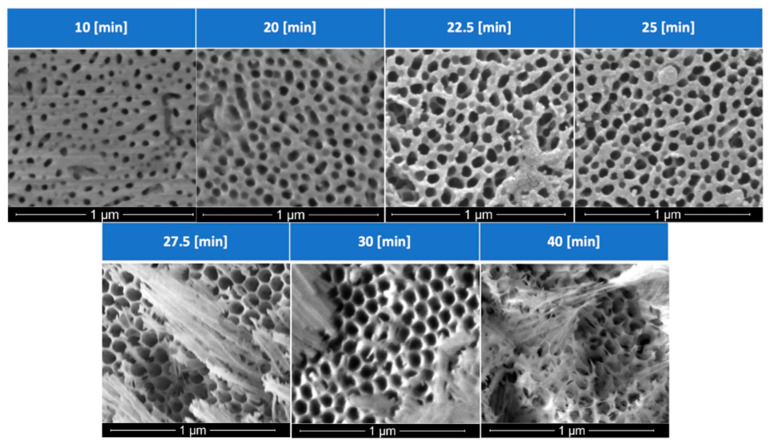
Effects of anodization times on the pore size and pore density of the anodic host layer.

The pores generated by diverse anodization conditions are presented in Figure 5 and Figure 6. Those SEM images show evidence of the regular arrangement of pores under most conditions employed. All samples in Figure 5 were fabricated by experiments that lasted 40 min at 40 °C in their respective concentrations. Larger pore volumes are observed for samples generated at 40 V when compared to those produced at 20 or 30 V. When looking at the oxalic acid concentration of 1.6 M, it was observed that higher voltages created larger diameter pore sizes in the anodic structure. For the application of phase change material impregnation, these larger pore sizes were preferred. Since the largest pore size shown in Figure 5 is observed at 40 V, such voltage and concentration were chosen to move forward with for the anodic layer development.

With the voltage and concentration of the solution fixed at 40 V and 1.6 M, respectively, time would not only determine the thickness of the anodic layer, but also the pore morphology. Figure 6 details the effects of anodization times: after 25 min under the mentioned experimental conditions, a more disordered and misoriented group of pores is generated. With an increase in porosity comes a thinning of the cell walls that surround each anodic pore, increasing the likelihood of columns that bend and break, blocking the anodic pores, rendering a microstructure ineffective for PCM impregnation, and reducing the ability to hold the PCM during thermal cycling.

**Figure 7:**
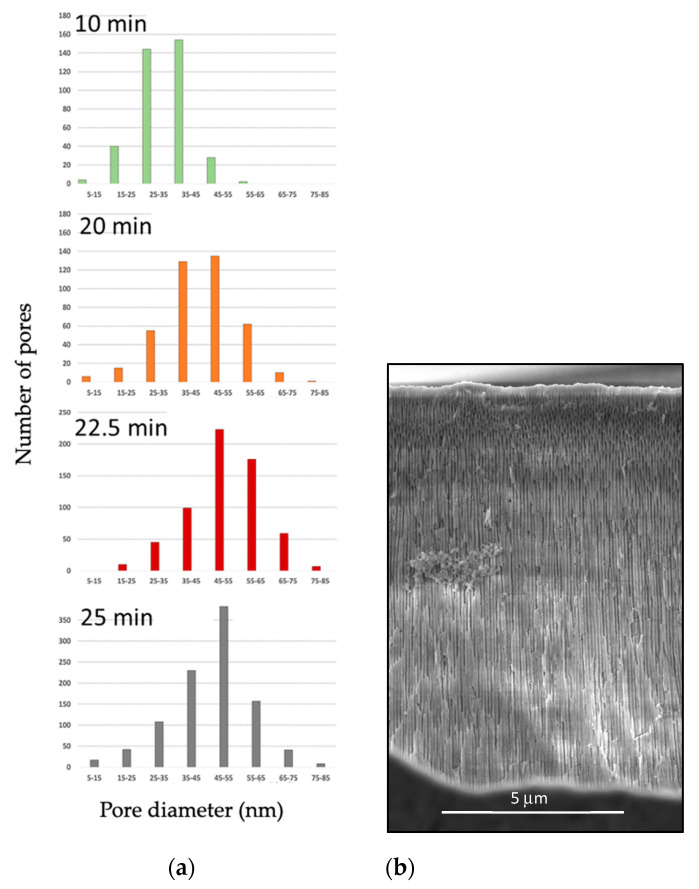
Pore size distribution analysis performed in (**a**) samples subject to diverse anodization times and (**b**) SEM cross–sectional image that illustrates pore length for the sample anodized 40 V, 1.6 M at 40 °C for 25 min.

A more detailed analysis of the effects of time on the pore size distribution is presented in Figure 7, where it can be observed that most pores have diameters in the 5–85 nm range, with average values that increase as the time of anodization is extended. The cross-section of samples was also analyzed. Figure 7 also presents a SEM micrograph representative of a cross-section of the sample anodized at 40 V, 1.6 M at 40 °C for 25 min, conditions that were selected for further annealing and thermal analysis. The pore length measurements were taken at 100 different locations, rendering an average length of 11.99 microns with a standard deviation of 0.64 mm. It is worth noticing that the method employed to section the sample left debris and fractured the porous channels in a path slightly different than the pore alignment.

Comparing these results with studies done in the past with anodic structures produced with oxalic acid, it is clear that there is a point at which overgrowth could occur. As temperature, current density, and electrolyte concentration increase, the rate at which the pores form is increased. As time continues to advance, the formation of the anodic layer becomes thicker, while the diameters of the pores will continue to grow larger. Those pores will eventually experience overgrowth or chalking. Chalking is due to a chemical attack on the outer part of the oxide film, which thins the pore walls and causes their upper regions to lose structural stability and collapse [15]. For future studies, the pore size could be further optimized to produce larger pore volumes and ease the introduction of PCM into the channels. Other work in the field that has demonstrated a high level of control in anodized structures includes [16,17,18].

Thermal treatment at 500 °C, conducted to enhance the mechanical robustness of the layer, did not cause significant changes in the microstructure of the samples, or morphology and size since SEM observations rendered diameters within the standard deviation of the measurements taken before annealing. The vacuum impregnation of the PCM in the anodic structures was followed by the application of a surface coat.

**Figure 8:**
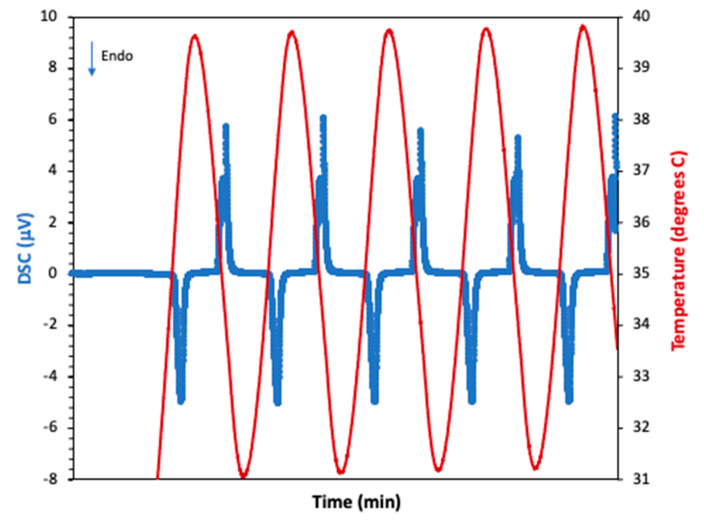
DSC analysis of eicosane showing the melting and solidification processes as the sample was cycled. The blue line represents the changes in the DSC signal (associated with the heat flow) and the red, the temperature.

**Figure 9:**
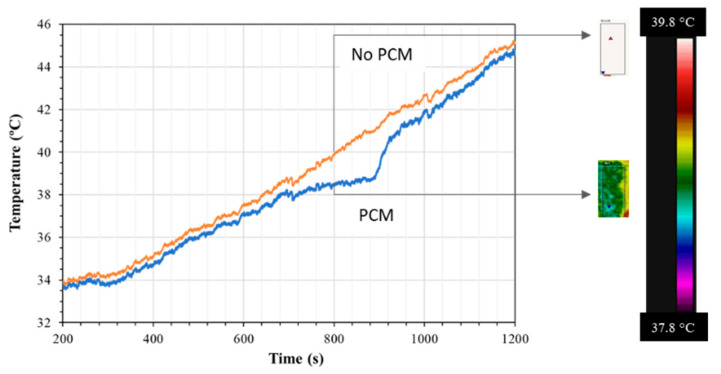
Surface temperature profiles detected by an IR thermal camera between samples containing an anodic structure that was annealed and sealed (orange line) and one that had the annealed anodic structure containing PCM and sealed (blue line). The thermal images along the scale bar of the FLIR are observed on the right side of the image.

As mentioned in the previous sections, the first test run was conducted with n-eicosane coated with Epofix epoxy. Figure 8 shows the thermal behavior of n-eicosane as it was cycled from 30 to 50 °C (5 cycles shown). The phase change experienced by the PCM produced the expected endothermic reaction during PCM melting (M.P. 36–38 °C), and an exothermic reaction during solidification. Figure 9 presents the temperature profiles of anodized, annealed aluminum samples tested as described in Section 2.1, with and without PCM. Tests that aimed to measure surface temperatures with an IR device showed that upon heating, this endothermic reaction slowed the rate of heating a significant amount. This effect lasted for approximately 4.88 min starting at a temperature of 37.06 °C and ending at a temperature of 39.02 °C. This resulted in a maximum temperature difference of 2.27 °C when compared with the anodic layer with an epoxy coat and no PCM, and a maximum temperature difference of 1 °C when compared with raw aluminum. Upon cooling, the exothermic reaction peaked at 36.96 °C, which is below the lower bounds of the PCM liquid phase transition temperature. During testing, however, the PCM leaked out of the epoxy coating, reducing the effectiveness of the sample to withstand repeated cycles.

The test performed with PCM sealed with two thin coats of silver paint presented a similar cooling effect to the one observed with Epofix; however, the silver paint coating was able to contain the PCM upon heating and cooling, a promising result regarding repeatability. The maximum relative temperature difference is 1.63 °C when compared with the anodic layer with the silver coat and no PCM, and a maximum temperature difference of 3.35 °C when compared with raw aluminum.

**Figure 10:**
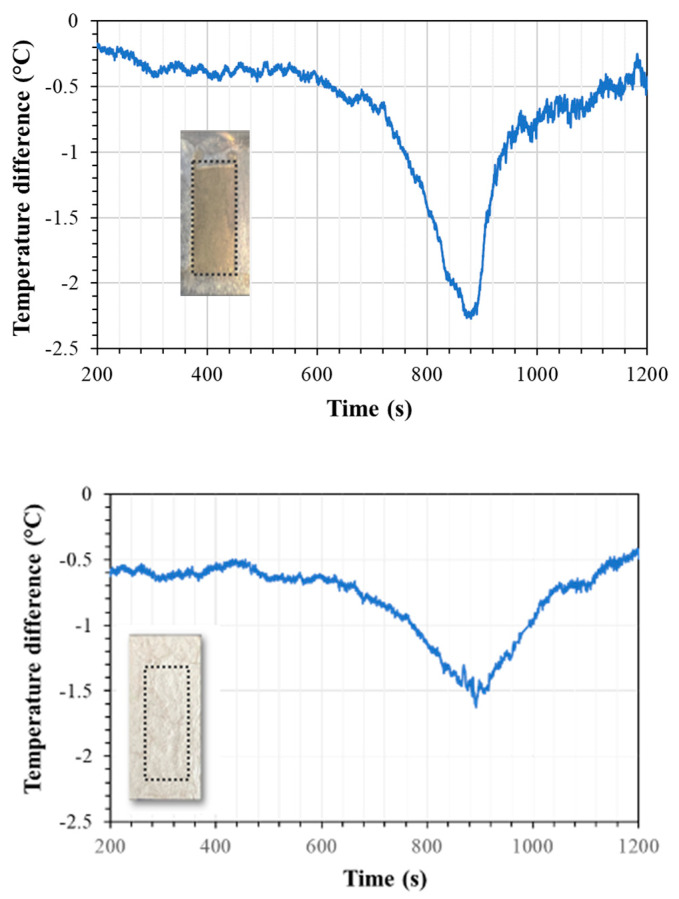
Surface temperature differences detected by a IR thermal camera between anodized samples with and without PCM that were sealed with epoxy resin (**top**) and silver paint (**bottom**). The images attached to the graphs show the areas where the anodic layer is located (dotted line). The epoxy coat is transparent, while the silver completely masks the location of the underlying composite structure.

Figure 10 presents the graphs of the temperature differences between metallic samples that had an anodic structure that was annealed and sealed and one that had the annealed anodic structure containing PCM and sealed with the Epofix coat (top) or silver paint coat (bottom) along with the images of the respective samples. Larger differences between the samples with and without PCM are detected for samples sealed with epoxy resin than those sealed with silver paint, although the latter seems to maintain a lower temperature during longer periods of time.

Other literature that encapsulates PCM in heat spreaders or similar structures reports larger reductions in peak temperatures than those observed here [2,19], however, in those cases, the metallic component employed had to be redesigned to host the PCM. From the observed results, it can be said that greater distribution of PCM with a conductor’s surface area allows the PCM to efficiently absorb heat from the conductor resulting in a net reduction of surface temperatures between 1 and 2.3 degrees C with only 0.05 g of n-eicosane was spread throughout a 120 mm^2^ section of aluminum. PCM within the anodic layer containing features in the nanometer scale increased the surface area contact of the PCM-conductor interface.

As a proof of concept, this study proved that the efficient application of the PCM within the microstructure can decrease the peak transient thermal loads of the system into which the PCM is integrated. Given the results presented herein, it is believed that latent heat energy storage could be achieved in more compact passive heat management devices, paving the way for technological advancement in the field of thermal management. Furthermore, PCMs incorporated within the surface microstructure of components could be applied not only to electronic devices but to machinery and other systems that operate under cyclic loads.

## 4. Conclusions

The goal of this study was to engineer a surface layer that incorporated PCM into a metallic part at the microstructural level through a simple postproduction process that could be applied to existing, geometrically complex components. An anodic layer was successfully fabricated on a given aluminum surface from an existing part. By adjusting the variables of electrolyte concentration, electrolyte temperature, time, and voltage, an anodic layer that contained pores with an average diameter of 50 nm was consistently fabricated for use with various PCMs and sealant methods.

The anodic layer was then annealed, a step that increased the mechanical robustness of the layer. Vacuum impregnation was successfully employed to incorporate PCM into the porous anodic structure and sealed with various coating agents. The vacuum impregnation chamber constructed as part of this work was highly effective at creating a vacuum seal. The metal-PCM composite generated withstood multiple heating cycles during the vacuum impregnation process, which was critical to the fabrication of multiple PCM-incorporated anodic samples.

The advantages of the approach presented herein are the simplicity of the strategy, the small amounts of PCM required, and the fact that there is no need to redesign the components. Moreover, anodization is a widely employed surface treatment and the process is easily scalable. The results indicate that there was a reduction of 1–2.3 degrees C in the peak temperatures that the metallic host experienced, with only 0.05 g of n-eicosane spread throughout a 120 mm^2^ section of aluminum, which is of itself a remarkable result for a composite structure that only measures a few microns of height and contains pores in the nanometer scale located in the surface of the component.
